# Serial Intervals for SARS-CoV-2 Omicron and Delta Variants, Belgium, November 19–December 31, 2021

**DOI:** 10.3201/eid2808.220220

**Published:** 2022-08

**Authors:** Cécile Kremer, Toon Braeye, Kristiaan Proesmans, Emmanuel André, Andrea Torneri, Niel Hens

**Affiliations:** Hasselt University, Hasselt, Belgium (C. Kremer, A. Torneri, N. Hens);; Sciensano, Brussels, Belgium (T. Braeye, K. Proesmans);; Katholieke Universiteit Leuven, Leuven, Belgium (E. André);; University Hospitals Leuven, Leuven (E. André);; University of Antwerp, Antwerp, Belgium (N. Hens)

**Keywords:** COVID-19, coronavirus disease, SARS-CoV-2, severe acute respiratory syndrome coronavirus 2, viruses, respiratory infections, zoonoses, vaccine-preventable diseases, serial interval, variants of concern, Belgium

## Abstract

We investigated the serial interval for SARS-CoV-2 Omicron BA.1 and Delta variants and observed a shorter serial interval for Omicron, suggesting faster transmission. Results indicate a relationship between empirical serial interval and vaccination status for both variants. Further assessment of the causes and extent of Omicron dominance over Delta is warranted.

The World Health Organization designated the SARS-CoV-2 Omicron BA.1 variant (B.1.1.529) as a variant of concern (VOC) on November 26, 2021 (*1*). Omicron shows a fast epidemic growth and has taken over as the dominant VOC from the previously dominant Delta variant (B.1.617.2) worldwide. In Belgium, the Omicron variant was the dominant circulating strain during December 27, 2021–January 9, 2022, identified in 88.5% of sequenced samples (*2*). The Omicron variant is more efficient at evading immunity, acquired from previous infection or vaccination (*3*,*4*), compared with the Delta variant. Another epidemiologic characteristic that may contribute to the rapid spread of Omicron is increased transmissibility, possibly attributable to an increase in the reproduction number or a shortened serial interval (i.e., the time difference between symptom onset in an infector and infectee) (*5*). In this study, we estimate the means and SDs of the serial interval for the Omicron and Delta variants and assess whether these variants are associated with different observed serial intervals. To gain more insights on the possible effects of vaccination, we also compare the observed serial intervals for different combinations of vaccination status in transmission pairs.

## The Study

Belgium has a contact tracing system in place, where COVID-19 confirmed case-patients are asked about their contacts from 2 days before symptom onset until 10 days after. We used genotype sequencing to detect variants. If a variant was found in a transmission chain, all case-patients belonging to that chain were assumed to be infected by that variant. We collected transmission pairs that could be linked either to Omicron or Delta infections in which the infector reported first symptoms during November 19–December 31, 2021. During this period, Omicron started to spread in Belgium and took over dominance from the Delta variant (*6*). The same nonpharmaceutical interventions were in place throughout this period; the stringency index (indicating the strictness of measures on a scale from 0 to 100) was 48. We assumed that the first confirmed case in a reconstructed transmission pair (i.e., the index case-patient) was the infector and the contact was the infectee. We excluded transmission pairs for which symptom onset was not available for either case, as well as pairs for which the observed serial interval was <−5 days or >15 days to ensure biologically plausible serial intervals (*7*,*8*). We assigned vaccination status to both cases in a transmission pair as unvaccinated (including partially vaccinated), vaccinated (i.e., completed vaccination cycle), and vaccinated plus booster (Appendix).

Of the 2,495 included transmission pairs, 86.61% were linked to transmission of the Omicron variant (Appendix Figure 1). We report the means and SDs of the observed serial intervals; the median was 3 days for all stratifications. All reported p values are based on a Mann-Whitney U test. We further stratified transmission pairs by household and vaccination status (Appendix Tables 1, 2).

**Figure 1:**
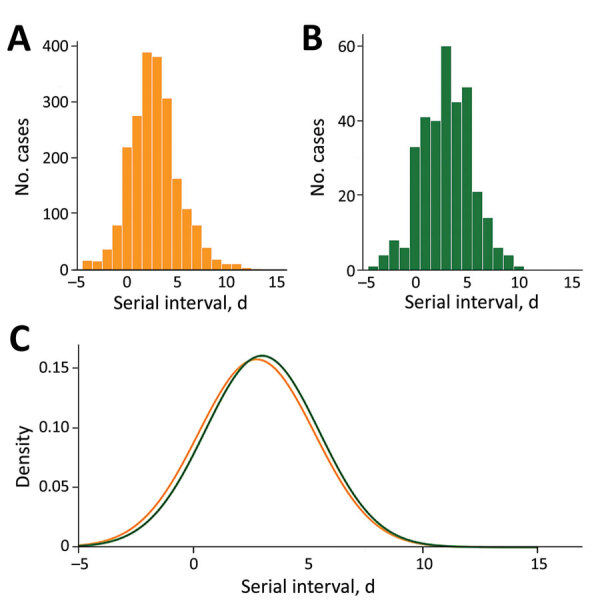
Empirical (A–B) and fitted normal (C) distributions of the serial intervals for SARS-CoV-2 Omicron and Delta variants, Belgium, for cases with onset date of infector during November 19–December 31, 2021.

The empirical serial interval distribution for Omicron had a mean of 2.75 days (SD 2.53 days), compared with 3.00 days (SD 2.48 days) for Delta (p = 0.019) (Figures 1, panels A, B). We estimated parameters of the normal distribution fit to both empirical serial interval distributions (Table; Figure 1, panel C). The empirical serial interval distribution for Omicron also was shorter than that for Delta within households (Table; Appendix Figure 2).

**Table:** Estimated parameters of a normal distribution for the serial interval of SARS-CoV-2 Omicron and Delta variants, by different stratifications, Belgium, November 19–December 31, 2021*

Variant	Stratification	No. transmission pairs	Posterior median (95% CrI)	SD (95% CrI)
Omicron	None	2,161	2.75 (2.65–2.86)	2.54 (2.46–2.61)
Delta	None	334	3.00 (2.73–3.26)	2.49 (2.31–2.69)
Omicron	Within-household	1,412	2.80 (2.67–2.93)	2.60 (2.50–2.70)
Delta	Within-household	278	3.04 (2.75–3.33)	2.43 (2.24–2.65)
Omicron	Between-household	672	2.72 (2.53–2.90)	2.44 (2.31–2.57)
Delta	Between-household	50	2.78 (2.00–3.56)	2.78 (2.30–3.45)
Omicron	Both unvaccinated	346	2.69 (2.40–2.98)	2.75 (2.55–2.96)
Delta	Both unvaccinated	61	2.54 (1.96–3.12)	2.29 (1.93–2.78)
Omicron	Both vaccinated	774	2.63 (2.46–2.81)	2.45 (2.33–2.58)
Delta	Both vaccinated	97	3.38 (2.89–3.88)	2.47 (2.16–2.86)
Omicron	Both vaccinated + booster	47	3.34 (2.58–4.10)	2.59 (2.13–3.24)
Delta	Both vaccinated + booster	0	NA	NA

*No Delta transmission pairs in which both case-patients had received a booster vaccine were reported. CrI, credible interval; NA, not available

**Figure 2:**
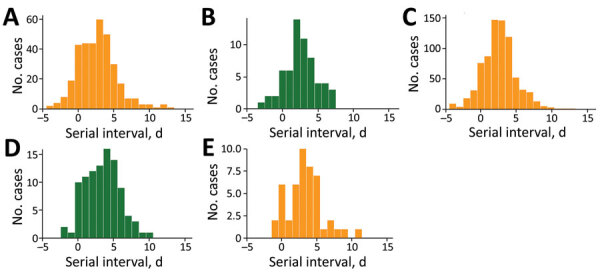
Empirical distribution of the serial intervals for SARS-CoV-2 Omicron (A, C, E) and Delta (B, D) variants, for transmission pairs where both cases are unvaccinated (A, B), vaccinated (C, D), or vaccinated with a booster (E, no data for Delta variant), Belgium, for cases with onset date of infector during November 19–December 31, 2021.

No difference in mean empirical serial intervals was found for pairs where both case-patients were unvaccinated or only partially vaccinated (2.69 vs. 2.54 days; p = 0.931) (Figure 2, panels A, B). For transmission pairs in which both case-patients were vaccinated (without booster), the mean empirical serial interval for Omicron was significantly shorter than that for Delta (2.63 vs. 3.38 days; p = 0.004) (Figure 2, panels C, D). The mean empirical serial interval for Omicron was longer for pairs that received a booster vaccine than for pairs that were vaccinated with only 2 doses (3.34 vs. 2.63 days; p = 0.065) (Figure 2, panels C–E). The mean empirical serial interval was significantly longer for the Delta variant in transmission pairs in which both case-patients completed the vaccination cycle, compared with those where both case-patients were unvaccinated or only partially vaccinated (3.38 versus 2.54 days; p = 0.045) (Figure 2, panels B–D).

## Conclusions

Our estimates of the empirical serial interval for Omicron are in line with those previously reported. Lee et al. (*9*) reported a mean serial interval of 2.8 days, and Kim et al. (*10*) estimated the mean serial interval to be 2.22 days (SD 1.62 days). Backer et al. (*7*) reported a mean serial interval of 3.5 and 3 days in 2 consecutive weeks for Omicron within-household pairs; in line with our findings, they found the interval to be shorter than that for Delta pairs. Shorter serial intervals suggest a possibly shorter generation time for the Omicron variant compared with Delta, pointing to faster transmission, which could explain the rapid growth that is observed for the Omicron variant. However, control measures and asymptomatic transmission may lead to different serial and generation interval distributions (*11*). Future studies estimating the generation interval for both variants are needed to shed more light on this matter.

The first limitation of our study is that self-reported symptom onset dates and contacts may be subject to recall bias. Likewise, the level of reporting contacts may differ for each person. We have used all reported transmission pairs, although some of them may have been wrongly assigned. We further assume that directionality of transmission was from index to contact, which may not be correct for each pair. However, because the same assumption was made for both variants, the comparison of serial intervals still holds, although serial interval lengths should be interpreted with caution. In addition, contacts were required to quarantine, which limited possible exposure from sources other than the reported index case-patient. We also do not explicitly account for right truncation (*12*); this choice is assumed not to affect our estimates because symptomatic infectees probably were not missed, given that we used the data available on January 17, 2022, but limited the serial interval to no more than 15 days. However, because contacts are reconstructed until 2 days before symptom onset of the index case-patient, possible left truncation might lead to exclusion of some transmission pairs. Selection bias attributable to targeted genotype sequencing of suspected Omicron cases (such as previously infected cases or travelers) might also have occurred, whereas genotype sequencing resulting in confirmed Delta cases might have been performed on samples from a hospital setting because severe disease was an indication for sequencing during the study period. This analysis does not correct for age or for previous infection; reinfections might be overrepresented among the Omicron cases.

Our results suggest that the empirical mean serial interval increases when both case-patients have a higher level of vaccine-induced immunity. However, possibly because of limited sample size, we did not observe this pattern for all possible combinations of vaccination status (Appendix Figure 3); more data are needed to properly assess the relationship between vaccination and serial interval. The empirical mean serial interval for unvaccinated and vaccinated (without booster) transmission pairs was similar for the Omicron variant. If vaccine-induced immunity and serial interval are positively correlated, this result might be explained by lower vaccine efficacy against Omicron for persons who have not yet received a booster vaccine. As the vaccination campaign progresses and more persons receive a booster vaccine, the reasons for and extent of Omicron’s dominance over Delta might need to be reassessed.

Appendix Additional information about serial intervals for SARS-CoV-2 Omicron and Delta variants, Belgium, November 19–December 31, 2021.
