# Supplementary material for: Serial Intervals for SARS-CoV-2 Omicron and Delta Variants, Belgium, November 19–December 31, 2021
Source: Emerg Infect Dis. 2022 Aug;28(8):1699–702. doi: 10.3201/eid2808.220220 (PMC9328897; doi:10.3201/eid2808.220220)
Supplement: Appendix — Additional information about serial intervals for SARS-CoV-2 Omicron and Delta variants, Belgium, November 19–December 31, 2021. [file 22-0220-Techapp-s1.pdf]

Article DOI: https://doi.org/10.3201/eid2808.220220

Serial Intervals for SARS-CoV-2 Omicron and Delta Variants, Belgium, November 19–December 31, 2021

Appendix

Detailed Description of Data and Analysis

During the study period, contacts that were considered high-risk (typically close contact (<1.5m) for longer time periods (>15 min)), were required to take a PCR-test. A first test was taken as soon as possible, a second test, when the first test was negative, was taken 7 days after the last contact. In this way, contacts that test positive for SARS-CoV-2 can be linked to a previously confirmed case and transmission pairs can be reconstructed. Vaccination was deemed to be effective after 7 days for Pfizer (second dose), 14 days for Moderna or AstraZeneca (second dose), 21 days for J&J (only 1 dose), or 7 days for a booster vaccine (Pfizer or Moderna). Other possible combinations seen as a complete vaccination schedule were 1 dose AstraZeneca followed by a Pfizer dose, or 1 dose Pfizer followed by Moderna. Household status was assigned based on information collected during the interview with the index case or provided by the healthcare worker.

We first analyze all transmission pairs for both the Omicron and the Delta variant, reporting the empirical distribution and computing the empirical mean and standard deviation of the observed serial intervals. Furthermore, we fit a normal distribution to the observed serial intervals, accounting for negative serial intervals, i.e., when the infectee shows symptoms before the infector (*1*). Normal distributions were fit using Markov Chain Monte Carlo (MCMC) methods and we report the posterior median and 95% credibility interval (CrI) for the mean and standard deviation. We then compared observed serial intervals for transmission that took place within and between households, where the latter refers to infection between individuals that do not live together. Furthermore, we compared observed serial intervals based on the vaccination

status of cases. The significance of differences in observed serial intervals was tested using a Mann-Whitney U test, since the diagnostics for a *t*-test were not met.

Reference

1. Ganyani T, Kremer C, Chen D, Torneri A, Faes C, Wallinga J, et al. Estimating the generation interval for coronavirus disease (COVID-19) based on symptom onset data, March 2020. Euro Surveill. 2020;25:2000257. PubMed https://doi.org/10.2807/1560-7917.ES.2020.25.17.2000257

Appendix Table 1. Proportion of within- and between-household transmission pairs for both variants

Household status	Variant	Proportion of all pairs
Within-household	Omicron	0.68
	Delta	0.85
Between-household	Omicron	0.32
	Delta	0.15

Appendix Table 2. Proportion of transmission pairs by vaccination status for both variants

Vaccination status	Variant	Proportion of all pairs
Both unvaccinated	Omicron	0.16
	Delta	0.18
Both vaccinated without booster	Omicron	0.36
	Delta	0.29
Both unvaccinated plus booster	Omicron	0.02
	Delta	0.00

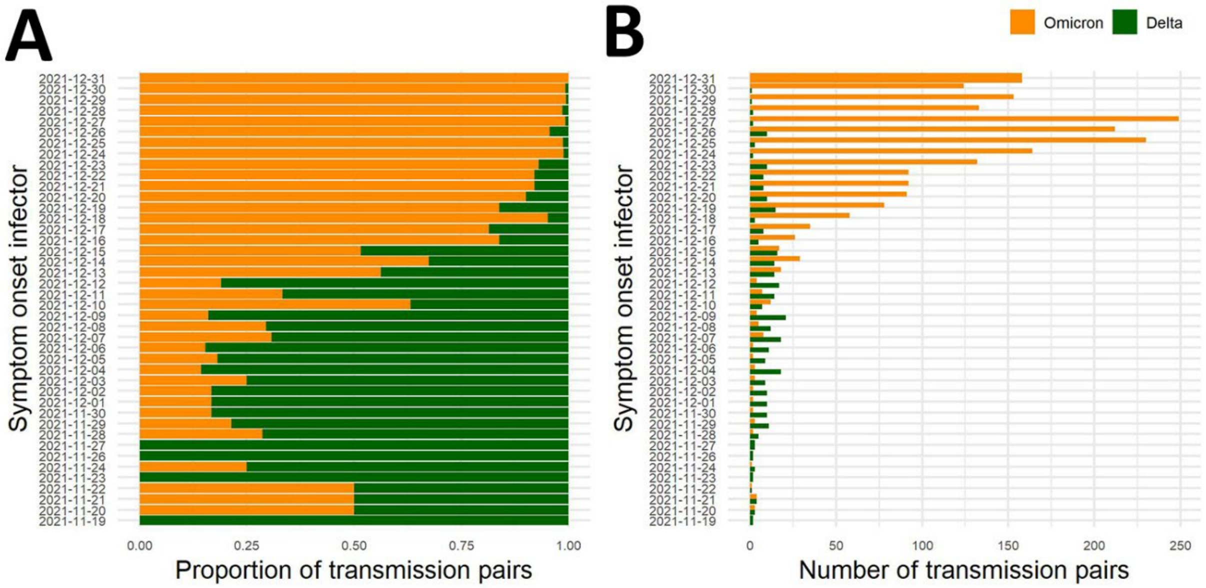

Appendix Figure 1. Proportion of all included (left) and total number (right) of transmission pairs linked to Omicron and Delta variant, by infector symptom onset date from 19 November to 31 December 2021.

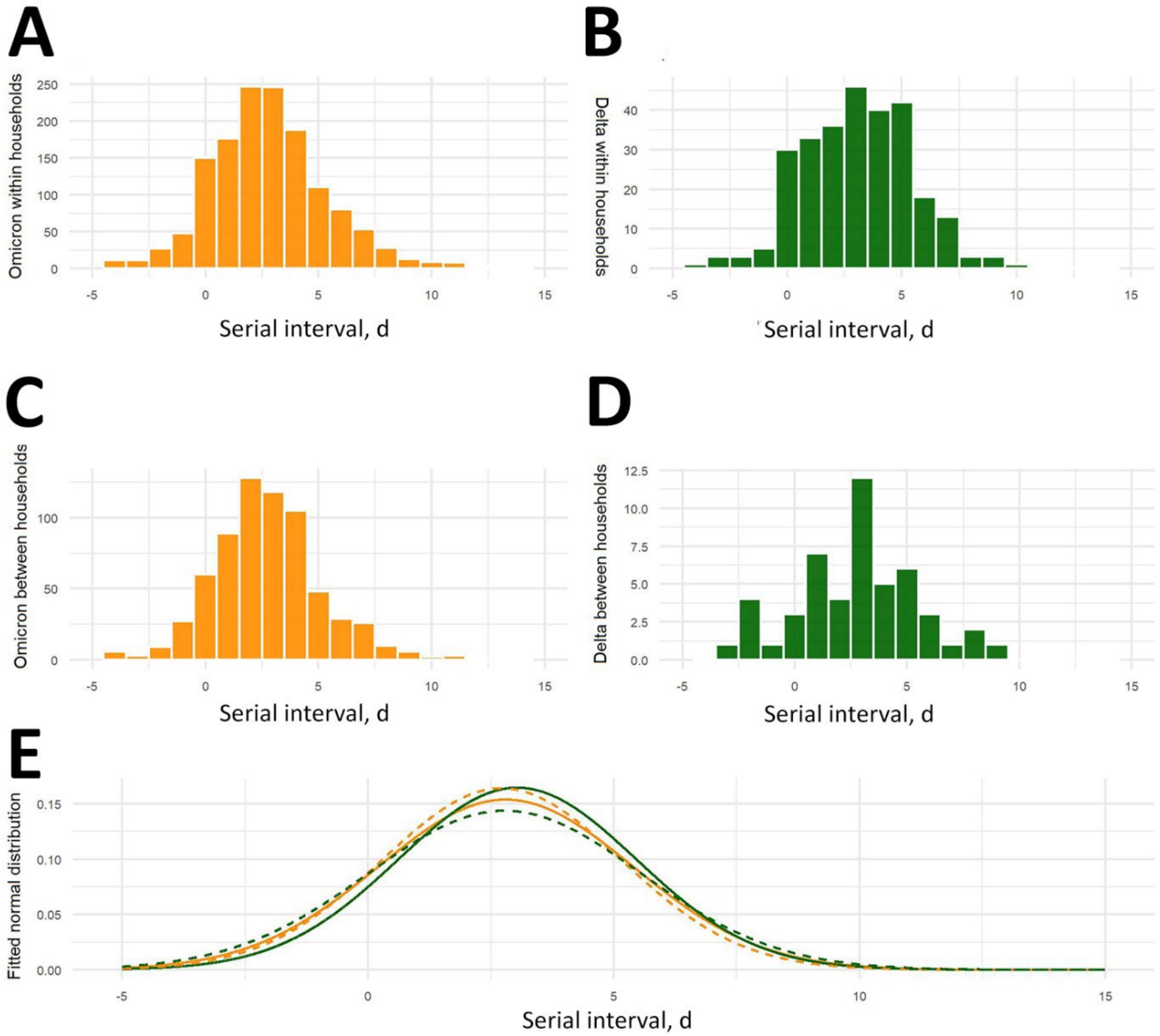

Appendix Figure 2. Empirical (A-D) and fitted normal (E; solid = within-household, dashed = between-household) distribution of the serial intervals for Omicron and Delta variant, by household status (excluding 83 pairs for which no information on household status was available). Onset date infector from 19 November until 31 December 2021. There was a significant difference between the mean empirical serial interval of both variants within households, but not between households (p = 0.034 for within-household, and p = 0.686 for between-household transmission pairs).

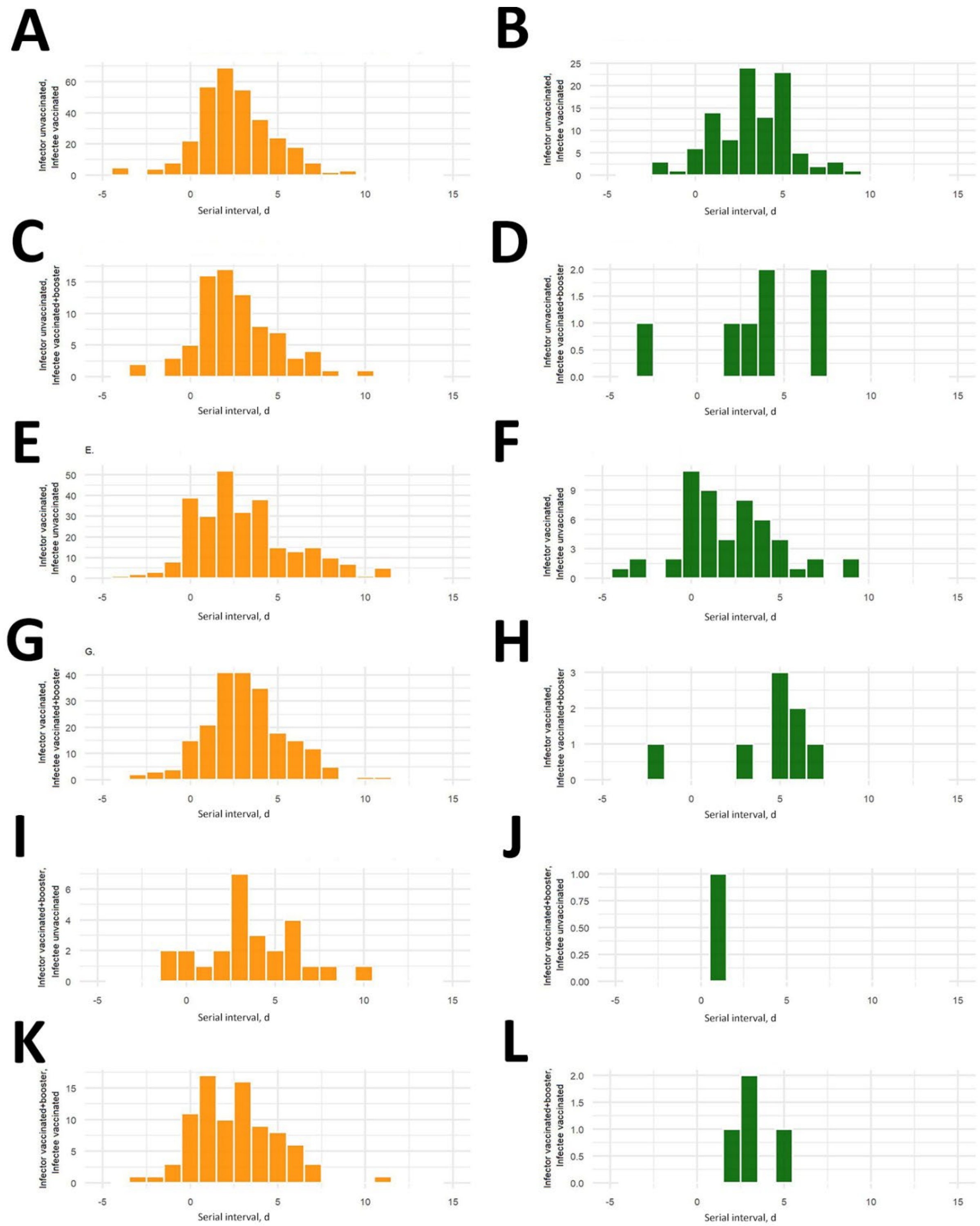

Appendix Figure 3. Empirical distribution of the serial intervals for Omicron (A, C, E, G, I, K) and Delta (B, D, F, H, J, L) variant, by vaccination status of infector and infectee. Infector symptom onset from 19 November until 31 December 2021.
